# Advanced strategy to produce insecticidal destruxins from lignocellulosic biomass *Miscanthus*

**DOI:** 10.1186/s13068-019-1530-8

**Published:** 2019-07-25

**Authors:** Ho Myeong Kim, In Seong Choi, Seoyoun Lee, In Min Hwang, Ho Hyun Chun, Seung Gon Wi, Jin-Cheol Kim, Tae Young Shin, Jong Cheol Kim, Jae Su Kim, Junheon Kim, Hae Woong Park

**Affiliations:** 1 R&D Division, World Institute of Kimchi, 86 Kimchi-ro, Nam-gu, Gwangju, 61755 Republic of Korea; 2 Asian Pear Research Institute, Chonnam National University, Gwangju, 61186 Republic of Korea; 3 Division of Applied Bioscience & Biotechnology, Chonnam National University, Gwangju, 61186 Republic of Korea; 4 Department of Agricultural Biology, College of Agricultural and Life Sciences, Chonbuk National University, Jeonju, 54896 Republic of Korea; 5 National Institute of Forest Science, Seoul, 02455 Republic of Korea

**Keywords:** Biorefinery, Destruxins, Pine wilt disease, *Miscanthus*, Biological control

## Abstract

**Background:**

Biorefineries are widely recognized as the most feasible solution to the problem of achieving environmental sustainability along with economic growth. Furthermore, pine wilt disease has caused severe environmental and economic damage worldwide to date. Herein, a highly efficient, advanced process for producing destruxins (DTXs) from *Miscanthus* (MCT) is reported, along with an application strategy.

**Results:**

The acetic acid–sodium chlorite pretreatment of MCT (AASC-MCT) is found to improve the monosaccharide production. Through biocatalytic conversion processes (simultaneous saccharification and cultivation), *Metarhizium anisopliae* JEF-279 can efficiently produce DTXs from 1% (w/v) AASC-MCT, i.e., DTX E (334.8 mg/L), A (288.8 mg/L), and B (48.6 mg/L). *Monochamus alternatus* (MA, Japanese pine sawyer) is known to act as a mediator transferring *Bursaphelenchus xylophilus* to pinewood. As *B. xylophilus* is associated with the occurrence of pine wilt disease, biological control of MA is a major strategy or controlling this disease. In this study, upon the application of a mixture of DTXs and protease-containing culture filtrate (PCF), complete mortality of MA is observed after a 5-day incubation. The MA immune system response is believed to cause an overexpression of actin and tropomyosin as a defense mechanism against the flaccid paralysis induced by the DTXs and PCF treatment.

**Conclusions:**

These results suggest that MCT can be used as a major feedstock in the biorefinery industry and that DTXs can be applied as an insecticide for biological control of pine wilt disease via MA termination.

**Electronic supplementary material:**

The online version of this article (10.1186/s13068-019-1530-8) contains supplementary material, which is available to authorized users.

## Background

The growing demand for chemical-based products constitutes a serious problem for the environment, as fossil fuels are used excessively to manufacture such products. However, biorefineries are widely recognized as the most feasible solution to the problem of achieving environmental sustainability, and they promote the growth of the bio-based economy [1]. A biorefinery is typically defined as an item of infrastructure in which sustainable biomass resources are converted into value-added materials using non-toxic heterogeneous catalysts [2, 3]. Based on recent market research, the market for global bio-based chemicals is expected to grow at a compound annual rate of 16.53% over the forecast period of 2018 to 2026 [4]. Therefore, various bio-based chemicals and value-added materials are being produced from a variety of biomass resources. In particular, *Miscanthus* (MCT) is a key biomass crop employed in the international biorefinery industry, because of its various advantages including its low-cost, seasonal-growth crop production [5].

Pine wilt disease severely damages both environments and economies worldwide, and the pinewood nematode *Bursaphelenchus xylophilus* is recognized as a major cause of this disease [6]. Chemical insecticides were previously used to control *B. xylophilus* but are now recognized as harmful substances that pollute soil, air, and water. In addition, overuse of chemical insecticides negatively affects the health of humans and other life forms [7]. *Monochamus alternatus* (MA, Japanese pine sawyer) is known to act as a mediator transferring *B. xylophilus* to pinewood [8]. Thus, biological control of MA is also required to prevent pine wilt disease from spreading.

Several previous studies have used proteases, essential plant oils, and chemical products to control nematode *B. xylophilus* [6]. However, biological control of arthropod pests using entomopathogenic fungi is a promising alternative. To date, entomopathogenic fungi such as *Metarhizium anisopliae* and *Beauveria bassiana* have been widely used as fungal agents for biological control of insect pests [9, 10]. In particular, *M. anisopliae* can kill hundreds of insect species and has been used to biosynthesize destruxins (DTXs), i.e., cyclic hexadepsipeptides composed of one α-hydroxyl acid and five amino acid residues [10, 11]. Sufficient DTXs infection can cause paralysis and death in insects because of its high toxicity [12]. In addition, DTXs exhibit a wide range of biological activities, including cytotoxicity, insecticidal activity, V-ATPase inhibition, anti-hepatitis B activity, and calcium channel blocking, with potential pharmaceutical applications in treating cancer, osteoporosis, and Alzheimer’s disease [10, 11].

This paper reports on an MCT biorefinery process for producing DTXs via biocatalytic conversion processes [simultaneous saccharification and cultivation (SSC)]. In addition, an advanced potential strategy for biological control of pine wilt disease through MA termination is suggested and evaluated.

## Results and discussion

Pine wilt disease has significantly damaged both the environment and economy in North America (the United States of America, Canada, and Mexico), Europe (Spain and Portugal), and Asia (the Republic of Korea, China, Japan, and Taiwan) (Additional file 1) [13]. This study produces DTXs from AASC-MCT and proposes an advanced potential strategy for biological control of pine wilt disease via MA termination.

### Chemical composition analysis and solid and sugar recovery of *Miscanthus*

Because of its complex structure of combined cellulose (43.4%), hemicellulose (23.8%), and lignin (23.5%), biorefining MCT requires pretreatment processes such as alkaline, inorganic salt, or hydrothermal pretreatments [14–16]. In particular, HPAC and AASC pretreatments are widely used to delignify lignocellulosic biomass to increase the enzymatic conversion yield [17–19].

**Fig. 1:**
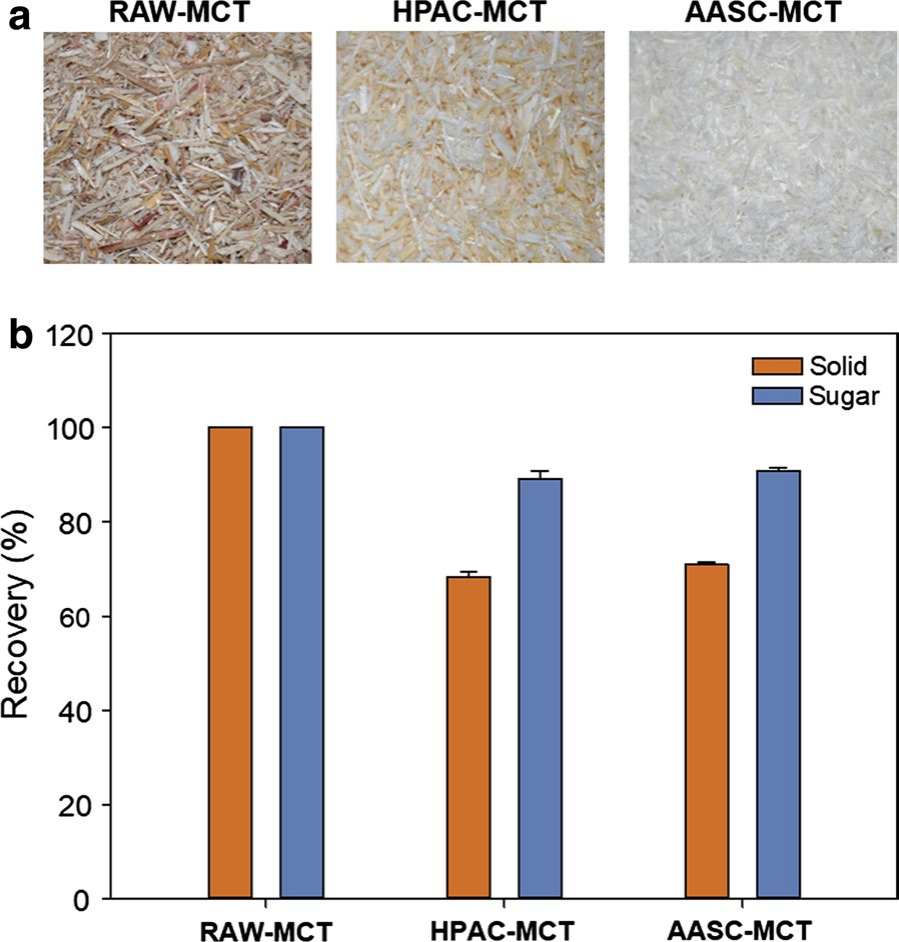
Changes in **a** phenotype and **b** solid and sugar recovery of MCT after pretreatment

In this study, we have analyzed the chemical composition, sugar, and solid recovery yield. According to the GC analysis, the monosaccharide compositions of the RAW-MCT, HPAC-MCT, and AASC-MCT mainly consisted of glucose and xylose (Table 1). In particular, the HPAC-MCT sample exhibited a high monosaccharide content (~ 88.5%). However, the AASC pretreatment more effectively improved the brightness than the HPAC pretreatment because of the efficient removal of lignin. Note that effectively removing lignin is essential, because this substance negatively affects the enzymatic hydrolysis [20]. After pretreating MCT with HPAC and AASC, the solid recoveries from the RAW-MCT were 68.3% and 71.0%, respectively, while the sugar recoveries were 89.3% and 90.9%, respectively (Fig. 1). Thus, the AASC pretreatment was slightly more effective for lignin removal, recovering more solids than the HPAC pretreatment (Table 1). However, there was no significant difference in sugar recoveries between AASC and HPAC pretreatments (*p* > 0.05).

**Table 1:** Chemical composition analyses of RAW-MCT, HPAC-MCT, and AASC-MCT

Dry matter (%, w/w)	Rhamnose	Arabinose	Xylose	Mannose	Galactose	Glucose	Total	Lignin
RAW-MCT	0.0 ± 0.0	1.9 ± 0.2	20.6 ± 1.1	0.0 ± 0.0	0.0 ± 0.0	45.2 ± 1.5	67.7 ± 2.7	23.5 ± 1.7
HPAC-MCT	0.0 ± 0.0	2.0 ± 0.1	23.4 ± 1.3	0.0 ± 0.0	0.0 ± 0.0	63.0 ± 2.7	88.5 ± 3.6	3.3 ± 0.8
AASC-MCT	0.0 ± 0.0	2.4 ± 0.3	23.2 ± 2.9	0.0 ± 0.0	0.0 ± 0.0	61.0 ± 1.6	86.7 ± 1.7	2.2 ± 0.8

Values represent the average over three replicates

### Enzyme optimization

The MCT cell-wall compositions consisted of cellulose, hemicellulose, and lignin [21]. Therefore, an enzyme cocktail was necessary for MCT degradation. In particular, xylanase is a key enzyme that accelerates the enzymatic hydrolysis of lignocellulose by removing hemicellulose [22].

In this study, we optimized the Viscozyme Wheat FG and pectinase enzyme loading content to decrease the enzyme volume. After enzymatic hydrolysis of the HPAC-MCT and AASC-MCT samples for 24 h, the sugar content was analyzed using HPLC, which revealed that glucose and xylose were mainly produced. Note that Viscozyme Wheat FG exhibits cellulase and xylanase functions and thus acts as the main enzyme for MCT degradation. The optimal enzyme loading contents were determined by the consideration of economic feasibility: 16.8 mg/g of Viscozyme Wheat FG and 6.2 mg/g of pectinase for HPAC-MCT; 8.4 mg/g of Viscozyme Wheat FG and 12.4 mg/g of pectinase for AASC-MCT. Hence, the soluble sugar concentrations (conversion yields) of the HPAC-MCT and AACE-MCT samples were 8.02 and 8.02 mg/mL (90.6 and 92.5%), respectively (Table 2).

**Table 2:** Sugar concentrations and conversion yields of HPAC-MCT and AASC-MCT hydrolysate under different enzyme loading contents

	Viscozyme wheat FG (mg/g MCT)	Pectinase (mg/g MCT)	HPAC, sugar (mg/mL)			Conversion yield (%)	AASC, sugar (mg/mL)			Conversion yield (%)
			Glucose	Xylose	Total		Glucose	Xylose	Total	
1	0	0	0.00	0.00	0.00	0.0	0.00	0.00	0.00	0.0
2	0	3.1	0.15	0.25	0.40	4.5	0.14	0.26	0.40	4.6
3	0	6.2	0.31	0.47	0.78	8.8	0.28	0.52	0.79	9.1
4	0	12.4	0.47	0.58	1.05	11.9	0.34	0.57	0.91	10.5
5	0	24.8	1.37	0.75	2.12	24.0	1.24	0.97	2.21	25.5
6	4.2	0	2.76	1.49	4.25	48.0	2.68	1.65	4.33	49.9
7	4.2	3.1	4.30	1.58	5.88	66.4	4.62	1.92	6.54	75.4
8	4.2	6.2	4.67	1.62	6.29	71.1	4.94	1.93	6.87	79.2
9	4.2	12.4	4.84	1.68	6.52	73.7	5.64	1.96	7.60	87.7
10	4.2	24.8	5.58	1.69	7.27	82.1	6.02	2.00	8.02	92.5
11	8.4	0	4.16	1.73	5.89	66.6	3.96	1.80	5.76	66.4
12	8.4	3.1	5.23	1.76	6.99	79.0	5.30	1.95	7.25	83.6
13	8.4	6.2	5.35	1.85	7.20	81.4	5.48	2.02	7.50	86.5
14	8.4	12.4	5.41	1.88	7.29	82.4	5.97	2.05	8.02	92.5
15	8.4	24.8	6.00	1.95	7.95	89.8	6.06	2.10	8.16	94.1
16	16.8	0	5.25	1.96	7.21	81.5	5.38	1.94	7.32	84.4
17	16.8	3.1	5.70	1.98	7.68	86.8	5.63	2.05	7.68	88.6
18	16.8	6.2	6.00	2.02	8.02	90.6	5.88	2.07	7.95	91.7
19	16.8	12.4	6.08	2.04	8.12	91.8	5.93	2.11	8.04	92.7
20	16.8	24.8	6.22	2.04	8.26	93.3	6.08	2.15	8.23	94.9

Values represent the average over three replicates

### Field-emission scanning electron microscopy

**Fig. 2:**
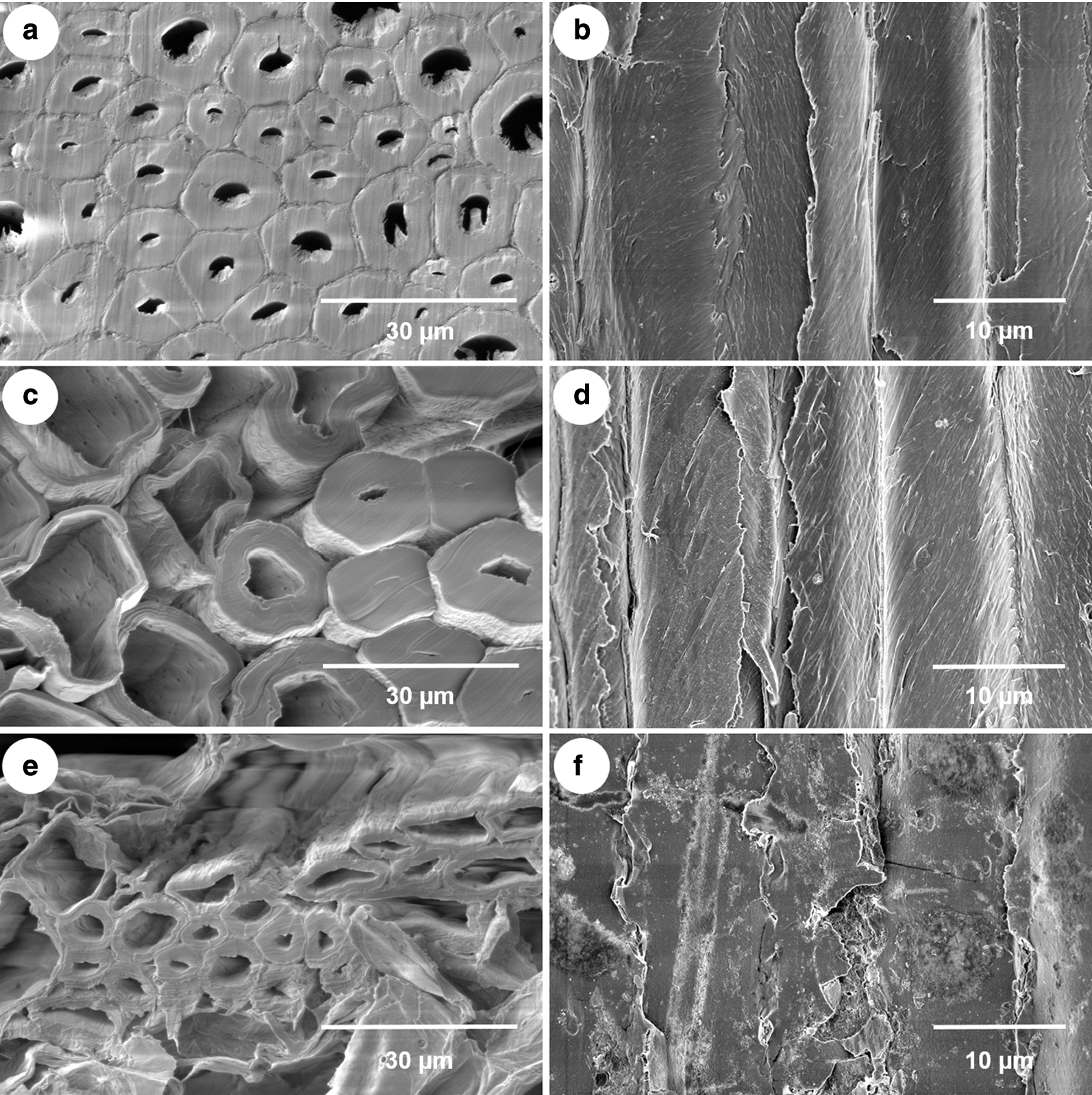
FE-SEM analysis results of MCT showing surface morphology changes after pretreatment. **a**, **b** RAW-MCT, **c**, **d** HPAC-MCT, **e**, **f** AASC-MCT

HPAC and AAPC pretreatments are well known as useful delignification methods [17–19]. As a complex organic polymer, lignin is a key structural material in lignocellulosic biomass cell walls that disrupts enzymatic hydrolysis [23]. After the pretreatments, we analyzed the physical changes in the RAW-MCT, HPAC-MCT, and AAPC-MCT samples using FE-SEM. Lignin removal via AASC pretreatment provided rougher surfaces and higher enzymatic accessibility than HPAC pretreatment (Fig. 2), both of which affected the enzymatic conversion and soluble sugar production.

### Analysis of relative crystallinity

MCT crystallinity is a major factor in monosaccharide production, because the high cellulose accessibility can increase the enzymatic hydrolysis yield [24]. In this study, the crystallinities of the RAW-MCT, HPAC-MCT, and AASC-MCT samples were analyzed via XRD. The XRD spectra for the RAW-MCT, HPAC-MCT, and AASC-MCT exhibited the characteristic peaks of cellulose Iβ at (1–10), (110), and (200) [25]. The CI values of the RAW-MCT, HPAC-MCT, and AASC-MCT were calculated to be 53.6%, 55.7%, and 61.2%, respectively (Additional file 2). Thus, the CI values were higher after pretreatment. Therefore, AASC pretreatment can efficiently remove amorphous MCT substances and improve the enzymatic conversion yield. In our previous study, pretreating coffee residue with AASC increased the CI value and enzymatic conversion yield by removing the phenolic and brown compounds and amorphous substances from the coffee residue [18].

### Destruxins production

For efficient DTXs production, SSC processes for RAW-MCT, HPAC-MCT, and AASC-MCT samples were preferentially investigated, because the SSC processes more efficiently produced DTXs than separate hydrolysis and cultivation (SHC).

**Fig. 3:**
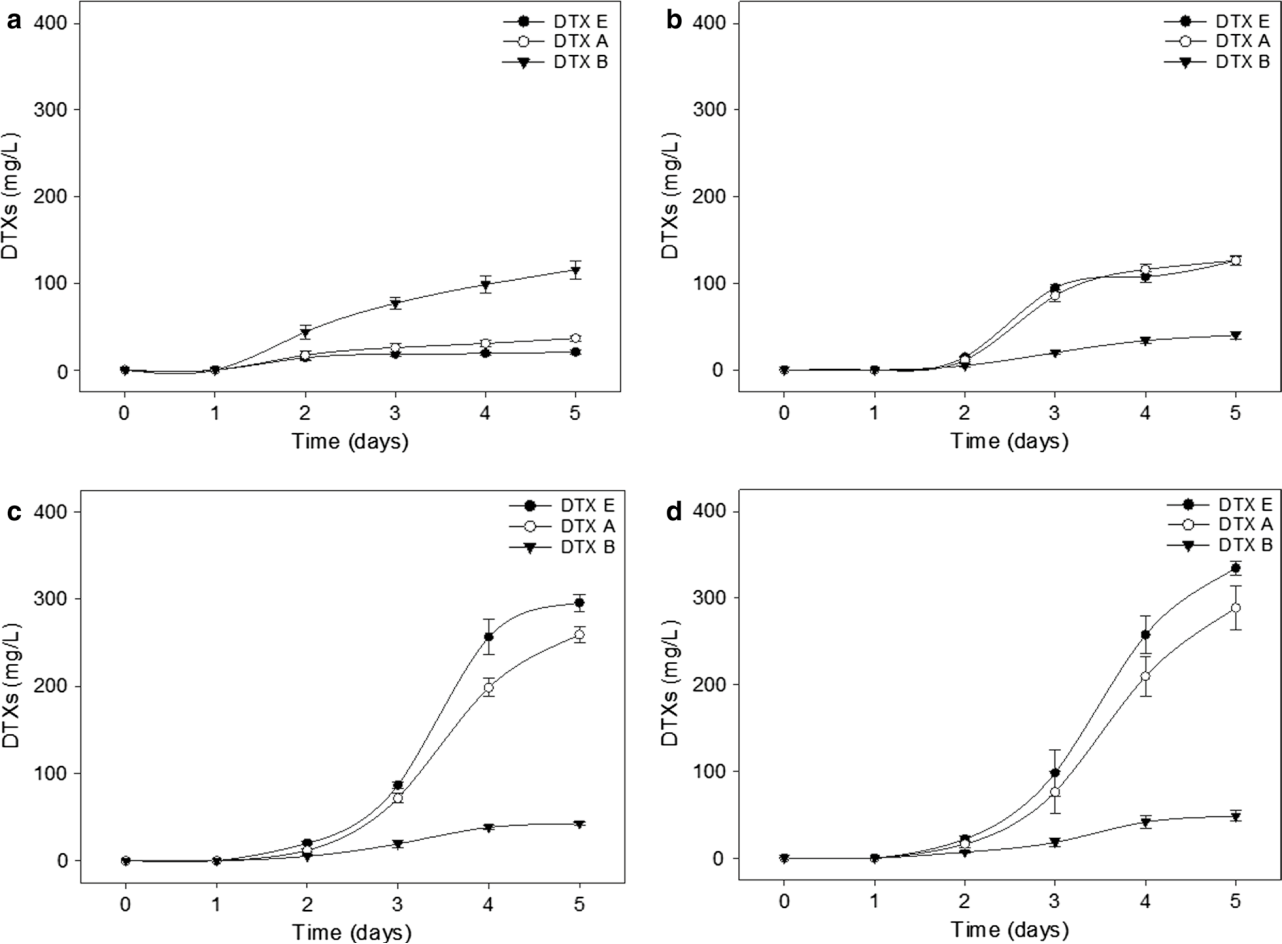
DTXs production of simultaneous saccharification and cultivation (SSC) processes according to carbon source. **a** Maltose 2.51% and glucose 0.43%, **b** RAW-MCT 1%, **c** HPAC-MCT 1%, and **d** AASC-MCT 1%

We compared the concentrations of DTXs produced at 32 °C for 5 days for each pretreatment condition. DTXs were produced on the second day of cultivation. After 5-day SSC processes, the RAW-MCT, HPAC-MCT, and AASC-MCT samples produced DTX E (126.0, 295.9, and 334.8 mg/L, respectively), DTX A (126.5, 259.3, and 288.8 mg/L, respectively), and DTX B (40.2, 42.8, and 48.6 mg/L, respectively) (Fig. 3 and Table 3). In a previous study, 2.51% maltose and 0.43% glucose were used as a carbon sources to produce DTXs [11]. This study used these same carbon sources to produce DTX E (20.7 mg/L), DTX A (36.6 mg/L), and DTX B (115.8 mg/L) (Table 3). These results indicate that *M. anisopliae* JEF-279 was more efficient, producing approximately 3.9-fold more DTXs from AASC-MCT than the pure substrate (2.51% maltose and 0.43% glucose). In addition, we produced DTXs with a substrate concentration of up to 5% (w/v) for the AASC-MCT samples. However, when the substrate concentration exceeded 2%, the fungus could not grow or produce DTXs. This is thought to have been due to approximately 27 mM acetic acid present in 2% (w/v) AASC-MCT sample, which has inhibited the fungus growth in this experiment. Similarly, the growth of a pathogenic fungus, *Colletotrichum* species, was inhibited by 30 mM acetic acid [26]. Therefore, the 1% (w/v) substrate concentration of AASC-MCT was found to be the optimal concentration for DTXs production.

**Table 3:** Summary of DTXs production according to SSC processing

Substrate	DTXs (mg/L)			Total (mg/L)	Yield (%)^a^
	DTX E	DTX A	DTX B		
Control (maltose 2.51% + glucose 0.43%)	20.7	36.6	115.8	173.1	0.59
RAW-MCT (1%)	126	126.5	40.2	292.7	4.32
HPAC-MCT (1%)	295.9	259.3	42.8	598	6.76
AASC-MCT (0.5%)	90.3	90.4	21.2	201.9	4.66
AASC-MCT (1.0%)	334.8	288.8	48.6	672.2	7.75
AASC-MCT (1.5%)	297.4	248.3	45.6	591.3	4.55

Values represent the average over three replicates

^a^Yields were calculated based on the initial sugar contents

### Insecticidal effect of culture filtrate on MA

Insecticides have various advantages, such as providing high-yield, high-quality crop production by killing many organisms. However, as insecticides are pesticides, they are highly toxic to humans and other life forms [7]. Control of pine wilt disease has become an urgent issue worldwide.

**Fig. 4:**
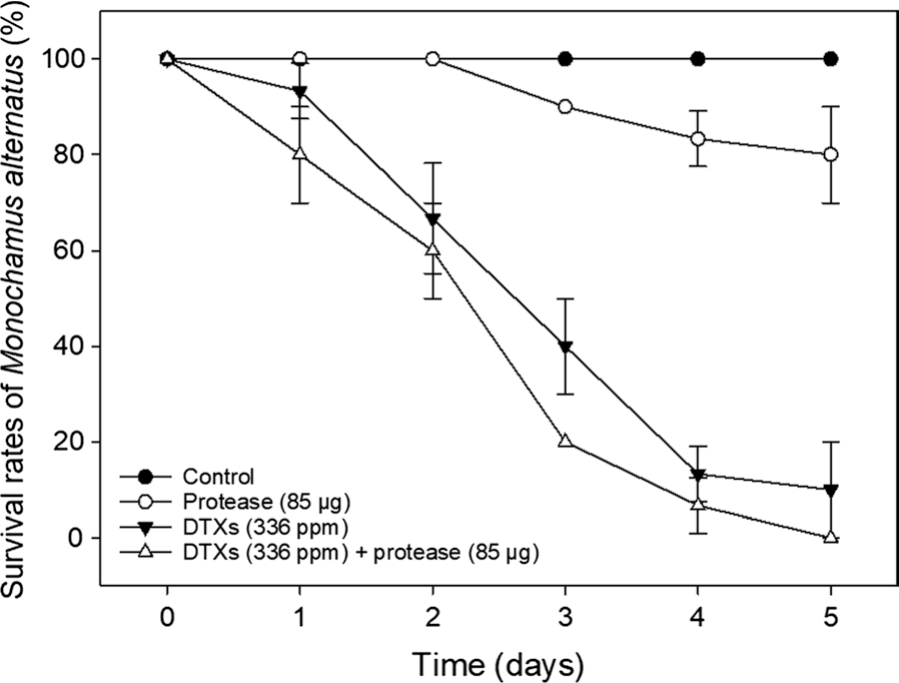
Insecticidal effects of DTXs and protease-containing culture filtrate on MA

The present study demonstrates a new strategy for biological control of pine wilt disease through high-efficiency MA termination. To evaluate the insecticidal effect of the metabolites produced from *M. anisopliae* JEF-279, four samples (sterile distilled water, protease-containing culture filtrate (PCF), DTXs, the mixture of DTXs and PCF) were sprayed on MA specimens. After 5-day incubations, complete MA mortality was confirmed for the sample subjected to the combined treatment of DTXs and PCF. The individual DTXs and PCF treatments of the MA yielded 90% and 20% mortality, respectively (Fig. 4). Consequently, the combined treatment involving both DTXs and PCF exhibits potential for biological control of pine wilt disease through MA termination. To the best of our knowledge, similar results have not been reported elsewhere to date.

### FE-SEM analysis of MA

**Fig. 5:**
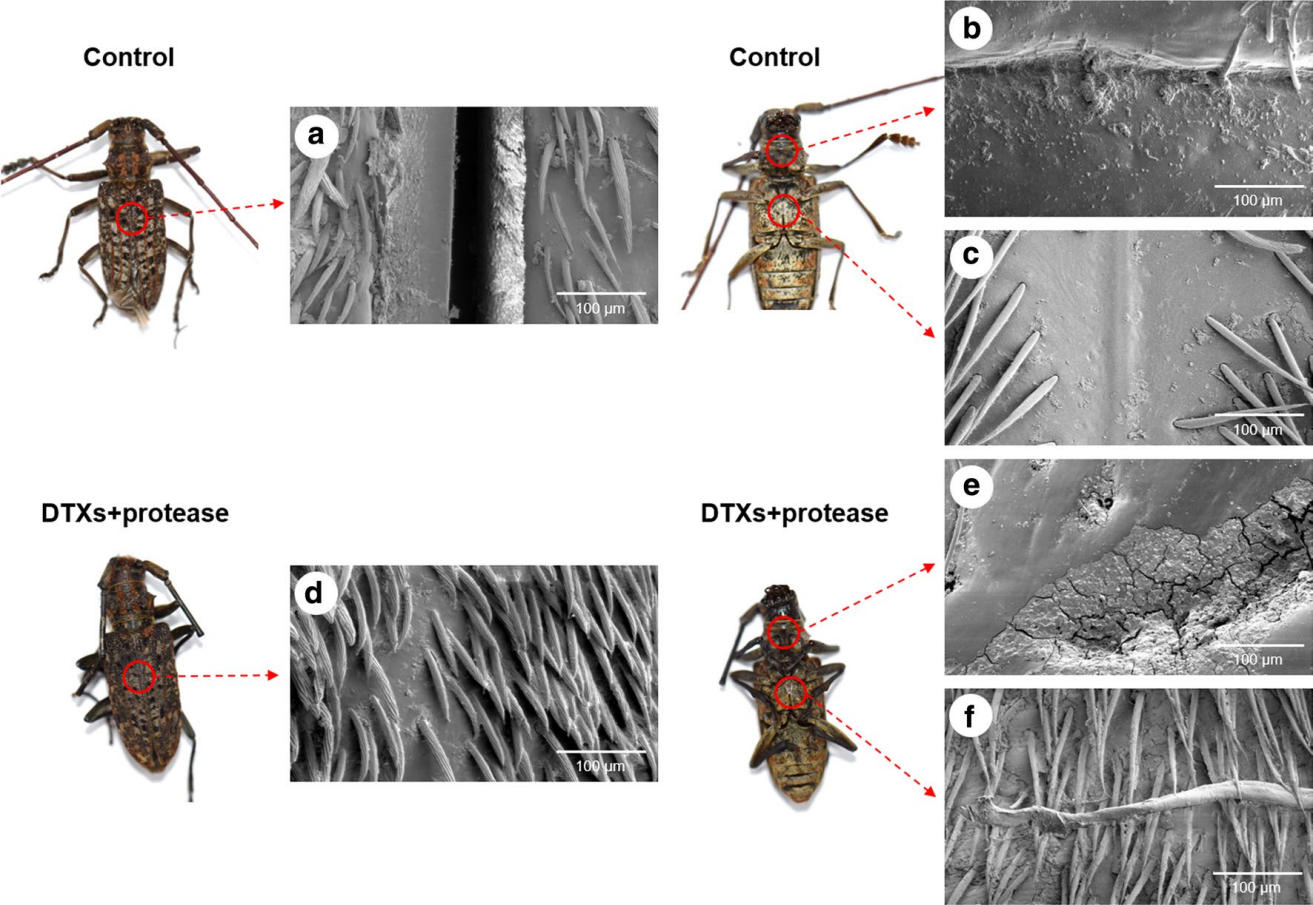
FE-SEM analyses of epicuticle changes in MA. **a** Back, **b** neck, and **c** thorax of RAW-MA sample; **d** back, **e** neck, and **f** thorax of DP-MA sample

Protease is a candidate insecticidal agent because of its toxicity to insects [27]. *M*. *anisopliae* produces cuticle-degrading enzymes such as protease, peptidase, and chitinase [28]. In particular, the subtilisin-like serine protease (PR1A) can rapidly hydrolyze the cuticle protein and facilitate cuticular penetration [27]. In this study, we observed changes in the epicuticle for the DP-MA sample after a 5-day treatment. FE-SEM analysis of the MA epicuticle before treatment revealed a rigid structure; however, after the mixed DTXs and PCF treatment, the epicuticle was heavily degraded in the MA neck and thorax after the mixed DTXs and PCF treatments (Fig. 5). These results indicate that PCF can be applied as a DTXs synergist for biological control of MA.

### Two-dimensional gel electrophoresis (2DE) and MALDI–TOF/TOF mass spectrometry analysis

**Fig. 6:**
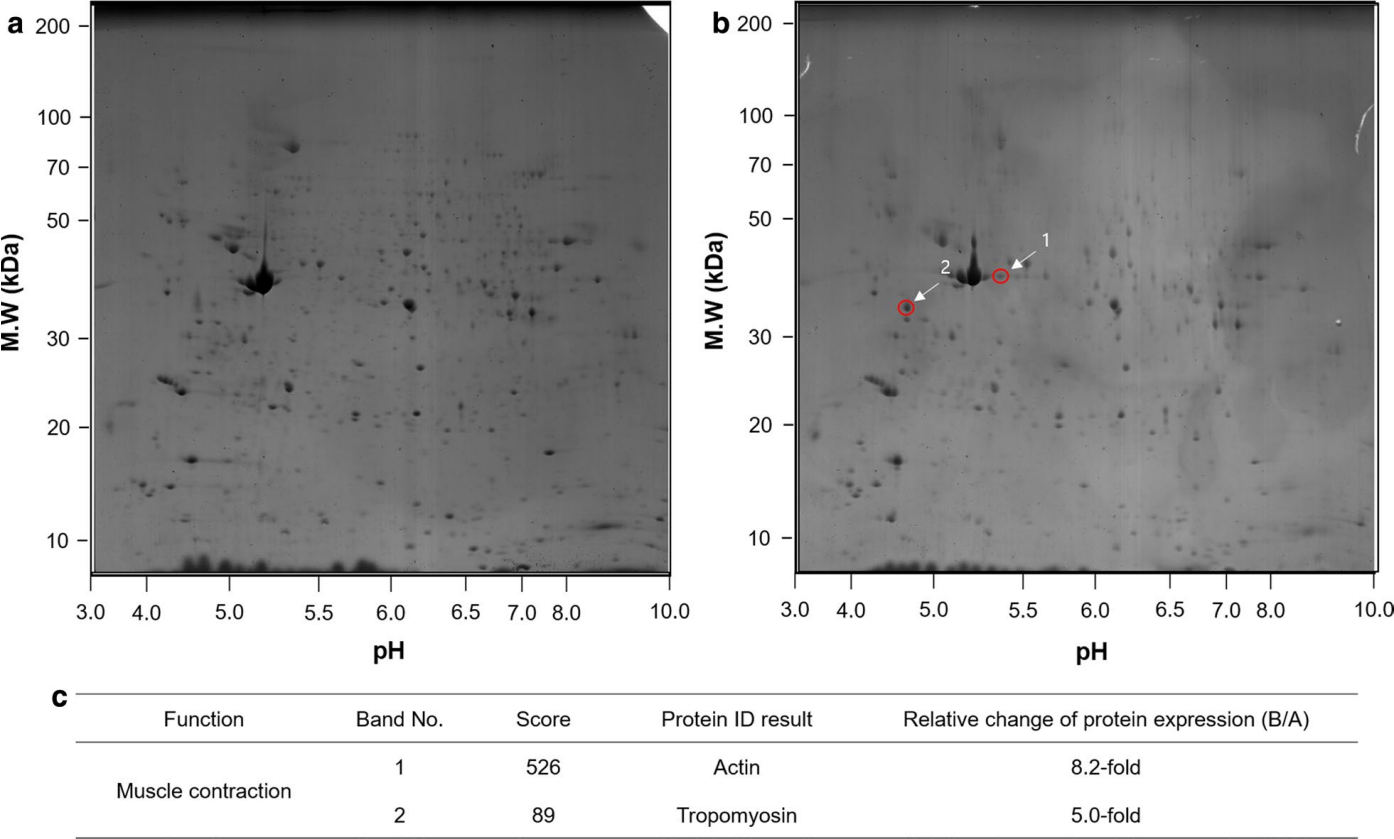
Analysis of two-dimensional gel electrophoresis (2DE) results for **a** RAW-MA and **b** DP-MA. **c** Proteins identified via MALDI-TOF/TOF mass spectrometry

To date, 2DE has been the primary proteomic analysis method because of certain advantages it provides, such as visualization and various kinds of information on protein spots, along with reliable evidence for existing protein isoforms [29]. Here, 2DE image analysis revealed approximately 676 and 349 protein spots in the RAW-MA and DP-MA samples, respectively (Figs. 6a, b, respectively). Among them, 309 paired and 407 un-paired protein spots were found. In the DP-MA sample, 80 protein spots were found to increase the protein expression level more than twofold, whereas seven protein spots decreased the protein expression level more than twofold compared to those in the RAW-MA sample.

Treating insects with DTXs insects causes tetanic paralysis and inhibits their defense response due to visceral muscle contraction, followed by flaccid paralysis [11, 30–32]. In this study, spray treatment of MA using a mixture of DTXs and PCF induced tetanic paralysis, followed by flaccid paralysis. To confirm this reaction, seven protein spots in the 2DE gel of the DP-MA sample were selected for protein identification. However, only two spots of the most expressed protein were identified because of the lack of an MA database. The expressions of protein spots 1 and 2 of the DP-MA sample (Fig. 6b) were increased by approximately 8.2- and 5.0-fold, respectively, compared to those of the RAW-MA (Fig. 6a), and they were interpreted as actin and tropomyosin, respectively (Fig. 6c). Actin (an abundant protein in eukaryotes) and tropomyosin are involved in muscle contraction [33, 34]. Thus, MA is believed to preferentially employ its immune system for muscle contraction by overexpressing actin and tropomyosin as a defense mechanism; this behavior arises in an attempt to overcome flaccid paralysis in response to treatment with DTXs and PCF.

## Conclusions

This study aimed to evaluate the possibility of producing DTXs from MCT and to propose the potential use of the produced metabolites as an insecticidal agent for biological control of pine wilt disease through MA termination. The SSC process can produce 0.33 g of DTX E, 0.29 g of DTX A, and 0.05 g of DTX B from 10 g of AASC-MCT. Additionally, combined treatment involving both DTXs and PCF yields complete MA mortality after a 5-day incubation. Under the above conditions, the MA overexpress actin and tropomyosin for muscle contraction as a defense mechanism. Therefore, we conclude that AASC-MCT can be a major natural feedstock for the biorefinery industry and that the produced metabolites may be applied in various biological fields and as insecticidal agents.

## Methods

### Biomass, pretreatment, and chemical composition

MCT was obtained from a *Miscanthus* field in Iksan, Republic of Korea, and then desiccated in a freeze dryer and stored at − 20 °C until further use. For the pretreatment, the MCT (10 g) was incubated with 100 mL of reagent (a mix of 50 mL of hydrogen peroxide and 50 mL of acetic acid, HPAC) at 80 °C for 2 h; then, the extraction was dried [17]. MCT (10 g) was also pretreated with acetic acid–sodium chlorite (AASC) using 4 g of sodium chlorite and 0.8 mL of acetic acid at 80 °C for 1 h [18]. This process was repeated 3 times/h by adding sodium chlorite and acetic acid, followed by drying the extraction. The chemical composition (holocellulose, Klason lignin) of raw and pretreated MCT was determined using TAPPI Standard Methods (1992) [35]. The monosaccharide contents of the raw, HPAC-pretreated MCT, and AASC-pretreated MCT were analyzed via gas chromatography (GC) and calculated based on the dry matters (%, w/w) [36].

### Enzyme optimization for fermentable sugar production

Viscozyme Wheat FG and Pectinex SP-L were purchased from Novozymes A/S (Bagsvaerd, Denmark). Enzymatic hydrolysis was performed on 1% substrate (HPAC-MCT and AASC-MCT, w/v) with variable loading contents of Viscozyme Wheat FG (0.0–16.8 mg/g MCT) and pectinase (0.0–24.8 mg/g MCT) for 24 h at 45 °C to confirm the optimal enzyme loading of the content. The monosaccharide content was determined via high-performance liquid chromatography (HPLC) using a refractive index detector (2414; Waters, Milford, MA, USA). A REZEX RPM (Phenomenex, Torrance, CA, USA) column (300 mm × 7.8 mm) was used to analyze the soluble sugar concentration at 85 °C, and the samples were eluted with deionized water at a flow rate of 0.6 mL min^−1^.

### Analysis of relative crystallinity

The relative crystallinity of the RAW-MCT, HPAC-MCT, and AASC-MCT samples was determined via X-ray diffraction (XRD) using a diffractometer with Cu-Kα radiation at 40 kV and 30 mA (X’Pert PRO MPD, PANalytical, Netherlands) [37]. The relative crystallinity values of the samples were recorded as the crystallinity index (CI) [38].

### Field-emission scanning electron microscopy (FE-SEM)

To facilitate field-emission scanning electron microscopy (FE-SEM), MA specimens treated with the RAW-MCT, HPAC-MCT, AASC-MCT, raw MA (RAW-MA), and DTXs and PCF (DP-MA) samples were fixed with a mixture of 2% (v/v) glutaraldehyde and 2% (v/v) paraformaldehyde in a 50 mM sodium cacodylate buffer (pH 7.2) at 4 °C for 4 h. After washing several times in a phosphate buffer, the samples were dehydrated using a graded ethanol series (50, 70, 90, 95, and 100%) and then dried with an HCP-2 critical point dryer (Hitachi, Tokyo, Japan). The samples were coated with gold and observed using FE-SEM (Helios G3 CX; FEI, USA).

### Destruxin production

*Metarhizium anisopliae* JEF-279 (KFCC11721P) was obtained from Chonbuk National University. To produce the DTXs, SSC processes were conducted in a total volume of 100 mL containing 1 mL of culture fluid of *M. anisopliae* JEF-279, 1% (w/v) dry matter (RAW-MCT, HPAC-MCT, or AASC-MCT), Viscozyme Wheat FG (8.4–16.8 mg/g MCT), pectinase (6.2–12.4 mg/g MCT), 0.75% bacto-peptone, and 0.02% β-alanine at 32 °C for 5 days. The DTX contents were analyzed using HPLC with an ultraviolet/visible light detector (2489; Waters) at 215 nm. A Kinetex^®^ 5-µm XB-C18 100 Å (Phenomenex, Torrance, CA, USA) column (250 mm × 4.6 mm) was used to analyze the DTX contents at 30 °C by adding a mobile phase at a flow rate of 1.0 mL min^−1^. The mobile phase corresponded to a linear gradient of deionized water and acetonitrile from 90:10 to 40:60 for 15 min, from 40:60 to 0:100 for 10 min, and from 100:30 for 5 min.

### Protease-containing culture filtrate production

*Metarhizium anisopliae* JEF-279 was cultured in a 500-mL flask containing 100 mL of Sabouraud dextrose broth at 200 rpm for 3 days. To produce the PCF, a 1% (v/v) seed culture was inoculated in a 5-L jar bioreactor (MARADO-05D-XS, BioCnS, Daejeon, Korea) in a total volume of 3 L containing 1% (w/v) wheat bran, 1% (w/v) soy protein, 0.42% (NH_4_)_2_SO_4_, 0.2% KH_2_PO_4_, 0.1% protease peptone, 0.02% urea, 0.03% CaCl_2_, 0.03% MgSO_4_, 0.2% Tween 80, and 0.2% trace element. *M. anisopliae* was cultivated at 28 °C for 5 days with agitation at 500 rpm and an aeration rate of 1.0 vvm (the volume of air added to the liquid volume per minute).

### Insecticidal effect of culture filtrate on *Monochamus alternatus* (MA)

To evaluate the insecticidal effect of the culture filtrate from *M. anisopliae* JEF-279, four samples (sterile distilled water, PCF, DTXs, and a mixture of DTXs and PCF) were prepared. Approximately 1 mL of each sample was sprayed on the MA specimens. The mortality was calculated from 30 MA for each sample, and three replications were conducted. Sterile distilled water was used as a negative control. The sprayed MA were stored in plate boxes (98 mm × 450 mm) at 28 °C in a 12–12 h, light–dark cycle. After every 24 h of incubation, the live MA were counted. The MA mortality rate was then calculated using Abbott’s formula [39]:

$$ \begin{aligned} {\text{Mortality }}\left( \% \right) \, = & \left[ {{{\left( {{\text{mortality percentage for treated sample}} - {\text{mortality percentage for untreated control}}} \right)} \mathord{\left/ {\vphantom {{\left( {{\text{mortality percentage for treated sample}} - {\text{mortality percentage for untreated control}}} \right)} {\left( { 100 - {\text{mortality percentage for untreated control}}} \right)}}} \right. \kern-0pt} {\left( { 100 - {\text{mortality percentage for untreated control}}} \right)}}} \right] \\ & \times \, 100. \\ \end{aligned} $$

### Analysis of 2DE and gel images

The proteins were diluted with rehydration solution (7 M urea, 2 M thiourea, 4.5% CHAPS, 100 mM DTE, 40 mM Tris; pH 8.8) and applied to immobilized pH 3–10 nonlinear gradient strips (IPG; Amersham Biosciences, Uppsala, Sweden) for two-dimensional gel electrophoresis (2DE) analysis. Isoelectric focusing was conducted at 80,000 Vh using an Ettan IPGphor 3 system (Amersham Biosciences). The second dimension was performed on 12% linear gradient polyacrylamide gels (18 cm × 20 cm × 1.5 mm) at a constant current of 40 mA for approximately 5 h. After protein fixation, the gels were stained with CBB G-250 for 12 h. The gels were then destained with H_2_O and scanned using a Bio-Rad (Richmond, CA) GS710 densitometer before being converted into electronic files. These files were then analyzed using the Image Master Platinum 5.0 image analysis program (Amersham Biosciences).

### Protein identification via matrix-assisted laser desorption/ionization (MALDI) time of flight (TOF)/TOF mass spectrometry (MS)

The peptide was eluted using 0.8 μL of a matrix solution (70% acetonitrile, 0.1% TFA, 10 mg/mL alpha-cyano-4-hydroxycinnamic acid). The eluted peptide was spotted on a stainless steel target plate. The peptide mass was then determined using a matrix-assisted laser desorption/ionization (MALDI) time of flight (TOF)/TOF mass spectrometer (4700 MALDI–TOF/TOF, Applied Biosystems), which was calibrated using the internal mass of trypsin. The peptide mass was analyzed according to the theoretical peptides of all proteins, based on the National Centre for Biotechnology Information database using the Mascot search program.

### Statistical analysis

Statistical analysis of data was conducted with the PASE software (ver. 17; SPSS Inc., USA). Analysis of variance (ANOVA) tests were used to determine the significant differences between treatments at *p* <0.05 using Tukey’s HSD test.

## Additional files

**Additional file 1.** Worldwide pine wilt disease distribution map.

**Additional file 2.** XRD profiles of RAW-MCT, HPAC-MCT, and AASC-MCT.

## Data Availability

The datasets used and/or analyzed during the current study are available from the corresponding author on reasonable request.

## Abbreviations

DTXs: destruxins
MCT: *Miscanthus*
MA: *Monochamus alternatus*
SSC: simultaneous saccharification and cultivation
SHC: separate hydrolysis and cultivation
AASC: acetic acid–sodium chlorite
HPAC: hydrogen peroxide–acetic acid
PCF: protease-containing culture filtrate
