# Supplementary figures and images for: Advanced strategy to produce insecticidal destruxins from lignocellulosic biomass Miscanthus
Source: Biotechnol Biofuels. 2019 Jul 25;12:188. doi: 10.1186/s13068-019-1530-8 (PMC6657178; doi:10.1186/s13068-019-1530-8)

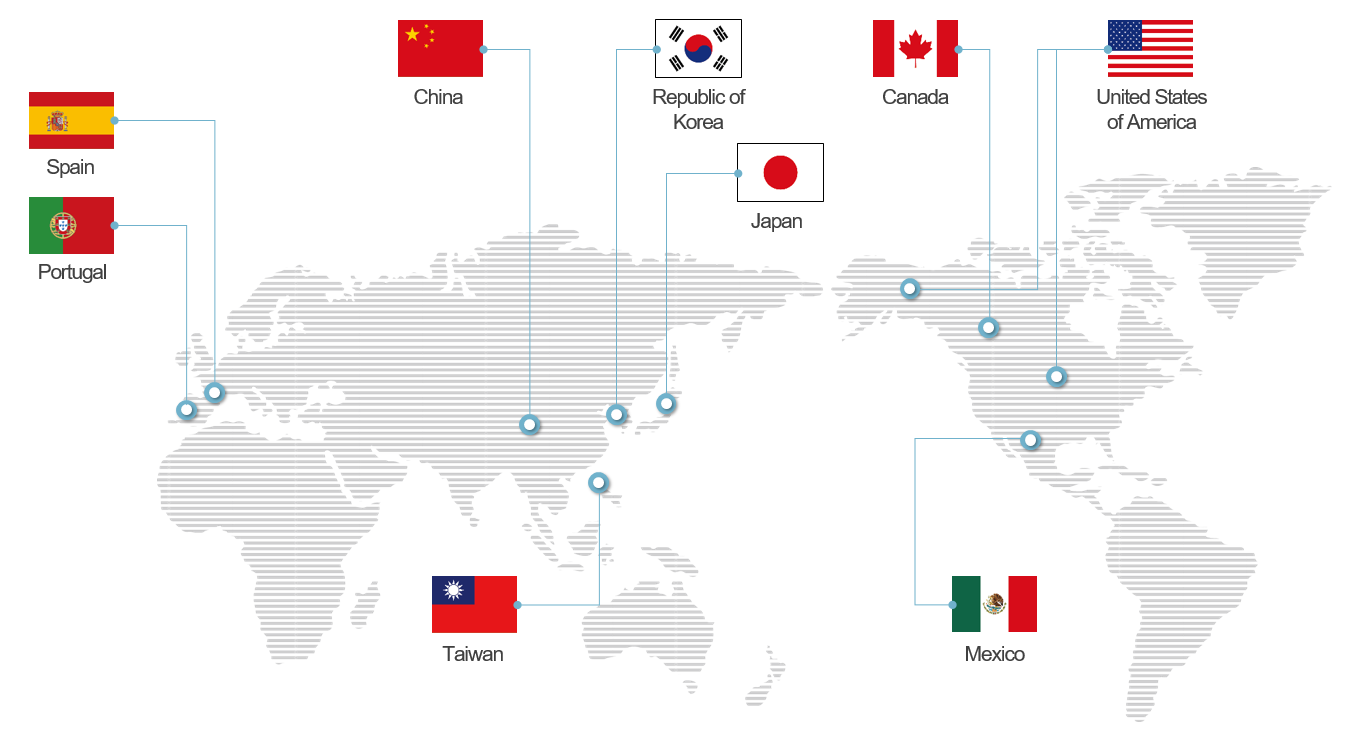

Supplement: Supplementary file 1 — Additional file 1. Worldwide pine wilt disease distribution map. [file 13068_2019_1530_MOESM1_ESM.tif]

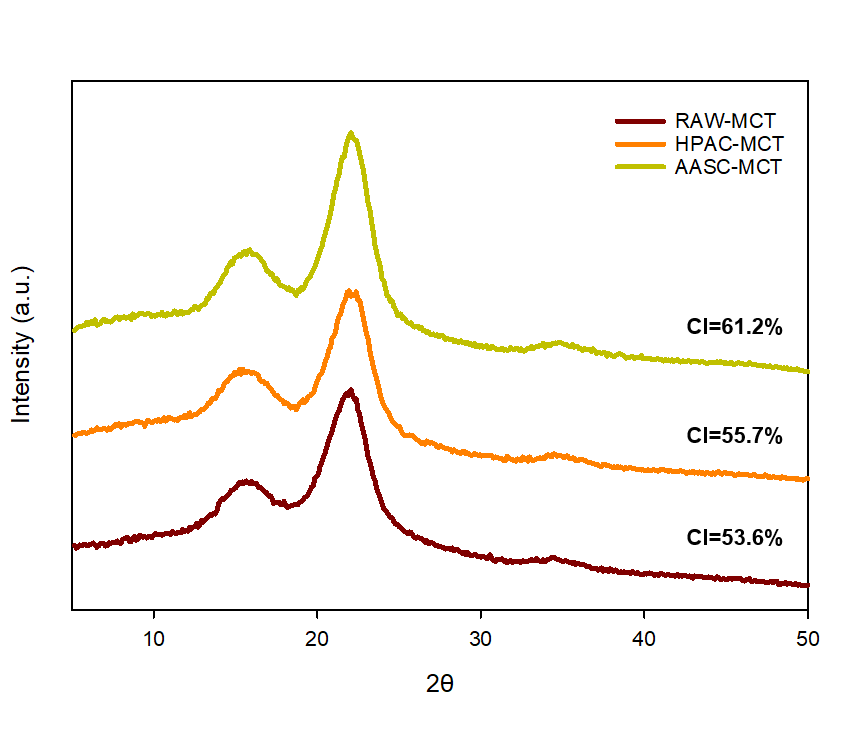

Supplement: Supplementary file 2 — Additional file 2. XRD profiles of RAW-MCT, HPAC-MCT, and AASC-MCT. [file 13068_2019_1530_MOESM2_ESM.tif]
